# Incorporating Refugia Capacity in Assessing Plant Climate Vulnerability: A Study Case on Alpine Endemics

**DOI:** 10.1002/ece3.72555

**Published:** 2026-02-03

**Authors:** Gabriele Casazza, Martino Adamo, Maria Guerrina, Luigi Minuto, Marco Mucciarelli

**Affiliations:** ^1^ Department of Earth, Environment and Life Sciences (DISTAV) University of Genoa Genoa Italy; ^2^ Department of Life Sciences and Systems Biology University of Torino Torino Italy; ^3^ Services Center for Hanbury Botanical Garden (GBH&HBG) University of Genoa Genoa Italy

**Keywords:** climate change, habitat suitability, holdouts, long‐term persistence, species distribution models, stepping stones

## Abstract

Anthropogenic climate change is a major threat to biodiversity in mountain ecosystems, particularly when hosting endemic species with limited ranges of distribution and low dispersal ability. These species may persist in situ if climatic conditions remain within their tolerance limits, or they can shift their range by tracking suitable habitats. We assessed the potential impact of global warming on seven endemic plant species, considering how different refugia types support species survival. Western Alps. We classified persistence areas into three categories: long‐term refugia (stable suitable areas across time), holdouts (temporarily suitable areas) and stepping stones (short‐term habitats facilitating range shifts). We used species distribution models to evaluate the potential impact of climate change on seven species, endemic to the South‐western Alps, examining how different refugia types support their survival and whether these patterns are species‐specific. We modelled habitat suitability under two climate scenarios (SSP245 and SSP585) across three future time slices, from 2021 to 2080, using five predictive models. Our results suggest that habitat suitability declines for most populations, but those of high elevation are less affected, often gaining suitability at higher elevations while losing it at lower ones. Long‐term refugia are the most common persistence type under moderate climate change, whereas holdouts dominate under extreme. Stepping stones are rare, suggesting that these species may struggle to track shifting climates. The distribution of refugia types follows an elevational gradient, with long‐term refugia at higher elevations and holdouts at mid‐elevations. Our findings highlight the vulnerability of Alpine endemics to climate change and emphasise the importance of conservation strategies that account for range shifts, also by active translocation, to ensure their long‐term survival.

## Graphical abstract

This study assesses the impact of climate change on seven endemic plant species in the Alps by modelling future habitat suitability under different climate scenarios. By identifying long‐term refugia, holdouts and stepping stones, the study reveals species‐specific patterns of persistence and highlights the limited potential for range shifts. The findings underscore the need for conservation strategies, including assisted migration, to support the survival of these vulnerable Alpine endemics.

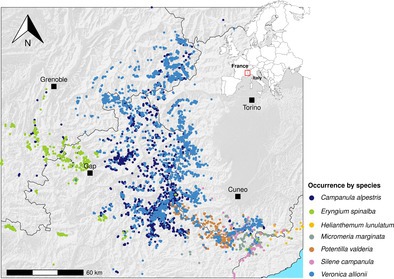

## 1 Introduction

Anthropogenic climate change is posing a serious threat to biodiversity, inducing short‐ and long‐term shifts in plant communities' composition and species' ranges (Montràs‐Janer et al. 2024). In general, under future climates, the ability of plant populations to survive in climatically suitable areas, within unfavourable surrounding climatic conditions, depends on the magnitude of climate alteration and on the species' tolerance limits. If the new climatic conditions fall within the tolerance limits of the species, they can persist in situ (Baumgartner et al. 2018), otherwise they can shift their distribution range to track suitable climatic conditions (Lenoir et al. 2008). However, not all species are able to successfully migrate; in fact, limited dispersal ability, physical barriers, or the absence of suitable habitats can hinder range shifts. In such cases, populations may decline and face local extinctions, ultimately compromising their long‐term survival. According to the level of buffering against future climatic conditions provided by an area in sustaining populations of a given species within its range, it is possible to recognise three different types of areas of persistence (Hannah et al. 2014; Keppel and Wardell‐Johnson 2015): (i) refugia, which allow long‐term persistence of populations in the same place; (ii) holdouts, in which the continuous deterioration of climatic conditions may enable populations to survive for only a limited duration, playing a role in facilitating the process of range shifts; (iii) stepping‐stones, populations growing in areas with favourable conditions of very short duration, but which are nevertheless close in space and time to future favourable areas, allowing individuals to follow climatic changes by gradually moving to areas that become favourable at different times. Furthermore, the possibility of plants to track suitable habitats is limited by their dispersal capacity (Morgan and Venn 2017). In fact, most plants disperse over short distances (Lososová et al. 2023) and thus may not be able to reach newly suitable areas regardless of distance. Current climate projections suggest a dramatic change from the current climate, which indicates that holdouts and stepping‐stones are more likely than refugia (Hannah et al. 2014). Conservation strategies should therefore be developed around the first two.

Climate change will impact all species and, in particular, range‐restricted species will be most negatively affected (Thuiller et al. 2006; Manes et al. 2021). Indeed, they are expected to have a narrow ecological niche, occurring in limited areas and specific habitats (Essl et al. 2009), and to be range‐limited because of dispersal limitations (Baselga et al. 2012) or constrained by topographic heterogeneity (Chytrý et al. 2024). Despite these limitations, persistence in situ and range shift by means of temporary climatically suitable areas may explain the survival of these species to past climate change despite their poor dispersal capability (Patsiou et al. 2014). Moreover, despite their rarity, range‐restricted species may play an important role in ecosystems. In fact, they usually support rare combinations of traits disproportionately increasing the potential breadth of functions provided by ecosystems (Mouillot et al. 2013).

Species distribution models (SDMs) are statistical tools that correlate species occurrence with environmental variables (Guisan and Zimmermann 2000) to estimate suitable areas for a species in different periods and/or regions (Araújo et al. 2019; Zurell et al. 2020). So far, they are the most widely used tools for assessing the effect of climate change on the distribution of species (e.g., Thuiller et al. 2011) and to detect areas where species would persist, disappear or migrate under future climate (e.g., Michalak et al. 2018). In particular, predicting where rare and endemic species may persist under future climates is crucial for planning protected areas in mountainous regions, creating ecological corridors across latitudinal and altitudinal gradients, minimising human‐induced pressures and ultimately planning reinforcing activities.

In this study, we used SDMs of seven range‐restricted plants endemic to Southwestern European Alps with different habitat preferences to evaluate the effectiveness of different refugia types in assuring survival of these taxa under future climate change scenarios. The main hypothesis behind the study is that the survival of range‐restricted plant species under future climate will be mainly favoured by holdouts and stepping‐stones more than stable refugia, especially in future harsher conditions.

More specifically, we are asking the following questions: (i) Are currently known populations threatened by climate change? (ii) What is the abundance of different types of persistence areas (i.e., refugia, holdouts and stepping stones)? (iii) What is the relative position of the different persistence areas with respect to the species' current distribution?

## 2 Materials and Methods

### 2.1 Study Area and Selected Species

**FIGURE 1:**
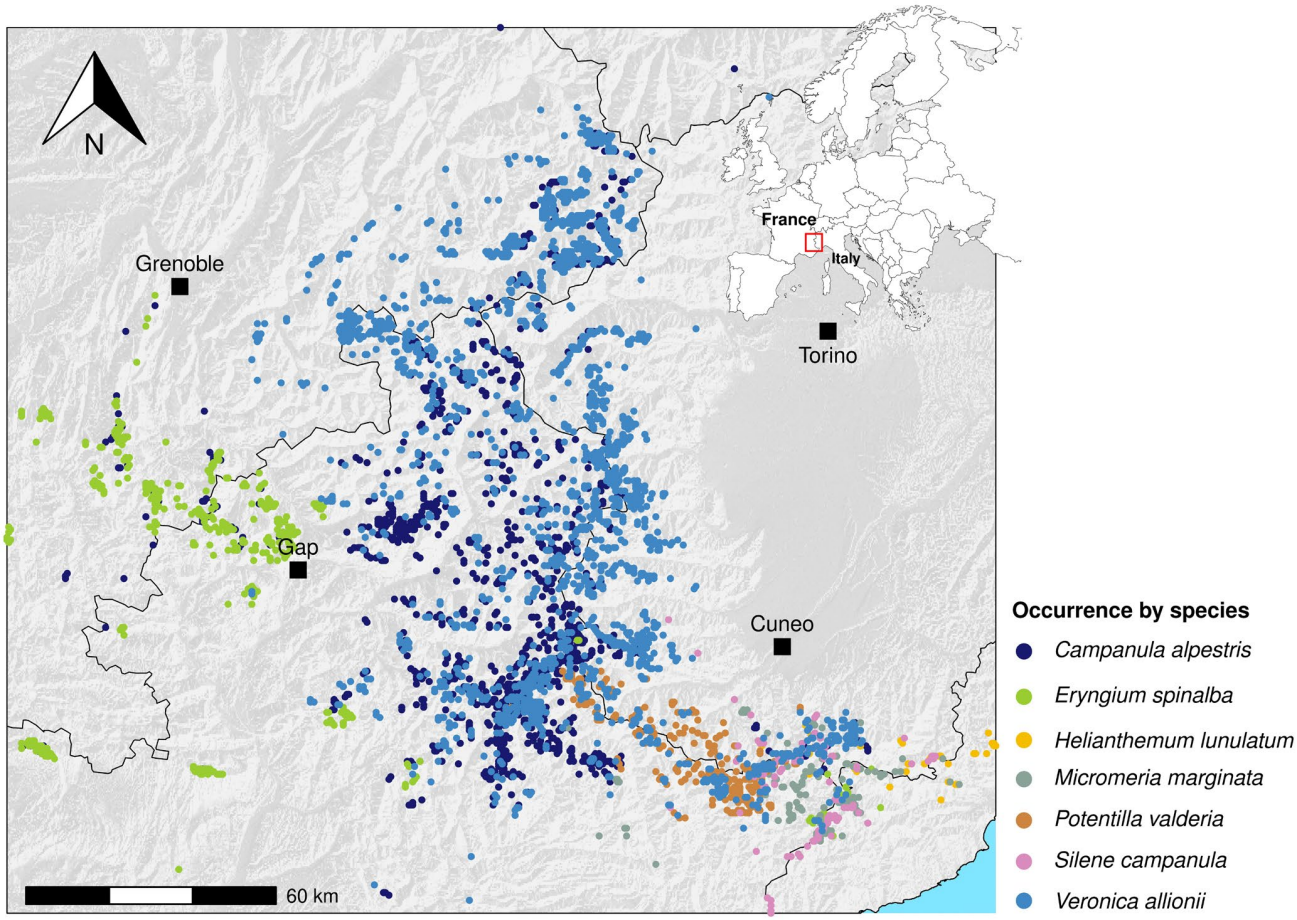
Location map of the study area. Occurrences are plotted on a hillshade map of the study area and different species are distinguished using the colour code given in the legend. Major towns in the area are indicated by a black square. On the top right, the study area is contextualised within Europe by a red polygon.

In this study, we selected seven range‐restricted species occurring in different habitats (Figure 1). *Potentilla valderia* L., *Veronica allionii* Vill. and *Eryngium spinalba* Vill. grow in grasslands; *Campanula alpestris* All. grows on fine debris and gravel; and *Helianthemum lunulatum* (All.) DC., *Micromeria marginata* (Sm.) Chater and *Silene campanula* Pers. grow on cliffs. *Helianthemum lunulatum* is listed as vulnerable in France; *C. alpestris* , *M. marginata* , *S. campanula* and *V. allionii* are protected by Italian legislation (national or regional); *H. lunulatum* and *E. spinalba* are protected by the legislation of both countries; and *P. valderia* is not protected. The mean elevation of *C. alpestris* , *V. allionii* and *P. valderia* is roughly 2200 m a.s.l.; *S. campanula* grows at the mean elevation of 1700 m a.s.l.; and *E. spinalba*, *H. lunulatum* and *M. marginata* are found at around 1500 m a.s.l. of elevation. Species were selected in order to represent all main ecologies of the endemisms from the southwestern Alps; moreover, we included only species with a highly trustable dataset of occurrences.

The occurrences' dataset was composed exclusively of GPS waypoints collected in the field extracted from regional databases (Conservatoire Botanique National SILENE database; http://www.silene.eu/index.php?cont=accueil and LiBiOss Regione Liguria; http://www.cartografiarl.regione.liguria.it/Biodiv/Biodiv.aspx), herbaria, literature and extensive field surveys made by the Authors or expert botanists. The final data set consisted of 4543 occurrences, ranging from 57 to 2083 occurrences per species in *H. lunulatum* and *V. allionii*, respectively.

### 2.2 Environmental Layers

We downloaded 19 bioclimatic variables for current (i.e., 1979–2013) and three future time slices (i.e., 2021–2040, 2041–2060 and 2061–2080) at about 1 × 1 km spatial resolution from the CHELSA v.2.1 data set (Karger et al. 2017). We have not included 2081–2100 climatic models due to the uncertainty already associated with these predictions (Gillett 2024). For the future climate, we chose two shared socioeconomic pathways (SSP) representing moderate and pessimistic possible future emission trajectories and coded according to radiative forcing (SSP245 and SSP585, 4.5 W m^−2^ by 2100 and 8.5 W m^−2^ by 2100, respectively). The SSP245 scenario represents the average path of future greenhouse gas emissions. This scenario assumes that climate mitigation measures are put in place. The SSP585 scenario represents the upper boundary of the range of climate scenarios. This scenario assumes that developing countries will grow following the trajectories of industrialised countries without relying on greenhouse gas efficient technologies. For each SSP, we used projections from three general circulation models (GCMs): MIROC6, MRI‐ESM2‐0 and UKESM1‐0‐LL. To assure the ability of models to make predictions in novel future environments (i.e., temporal transferability), we used the first two axes of a principal component analysis (PCA) as environmental variables for species distribution modelling, as suggested by Petitpierre et al. (2017). The PCA was calculated on all bioclimatic rasters of the study area pooled together, then the values of the first two axes of the PCA of each climate were separated (i.e., all the combinations of SSPs and GCMs).

Because using a coarse scale could underestimate the potential impacts of climate change on mountain plants, without taking into account microclimatic conditions, we statistically downscaled the climate predictors to a resolution of 300 × 300 m. In short, we resampled through Nearest Neighbour a DEM at 300 m resolution, from the 30 m Copernicus DEM. We calculated slope and aspect, the latter as northness [cos(aspect)] and eastness [sin(aspect)], by using the terrain() function in the R package ‘raster’. We included an irradiation predictor (Direct Normal Irradiation, DNI [kWh/m^2^]) from https://www.esmap.org/re_mapping. We calculated the geographical distance to the sea coastline by using the dist2Line() function in the R package ‘geosphere’. Then, we interpolated the PC1 and PC2 climatic predictors on the six physiographic variables at 300 m (elevation, slope, northness, eastness, DNI, sea distance) following the method of Geographic Weighted Regressions as in Lenoir et al. (2017).

### 2.3 Species Distribution Modelling

To account for model‐based uncertainties in the modelling process (Araújo and New 2007), we used five species distribution modelling techniques implemented in the R package ‘BIOMOD2’ v 4.2.1 (Thuiller et al. 2009) belonging to three different model families: two machine learning methods (i.e., generalised boosted models—GBM and Maximum Entropy—MaxNet), two regression methods (i.e., generalised linear models—GLM, and multivariate adaptive regression splines—MARS) and one envelope‐style method (Surface Range Envelope—SRE). We used the ‘BigBoss’ option to automatically tune each algorithm with the best parameters. To mitigate pseudo‐replication of occurrences, we retained for each species only one occurrence per grid cell. We randomly generated 10 sets of pseudo‐absences for species, each with 10,000 locations. For each pseudo‐absence set, observations were randomly split into two data sets, with 70% of the cells selected for training and the remaining 30% selected for model evaluation; we repeated 10 times a split‐sample cross‐validation. Model performance was assessed by using two different measures implemented in BIOMOD2: the area under the curve (AUC) of a ROC plot (Hanley and McNeil 1982) and the true skill statistic (TSS; Allouche et al. 2006).

### 2.4 Climatic Refugia Types

Using the SDM approach, we calculated habitat suitability values for each 300 × 300 m cell for each species across four time slices (1979–2013, 2021–2040, 2041–2060 and 2061–2080). Habitat suitability values range from 0 to 1 and indicate the predicted likelihood of a species occurring in a given cell based on environmental conditions. Values approaching 1 correspond to highly suitable habitats, whereas values near 0 indicate unsuitable conditions.

Refugia were modelled as cells with habitat suitability higher than 0.5 for the species over all three future time slices; thus they represented small islands of climatically favourable habitats within regionally deteriorating conditions. Holdouts were defined as cells with stable high habitat suitability (> 0.5) for the species over two out of three future time slices. Stepping‐stones were defined as cells with habitat suitability values > 0.5 that did not correspond to the current occurrences, but were immediately adjacent to them, and subsequently adjacent to other highly suitable cells across successive future time slices.

## 3 Results

**FIGURE 2:**
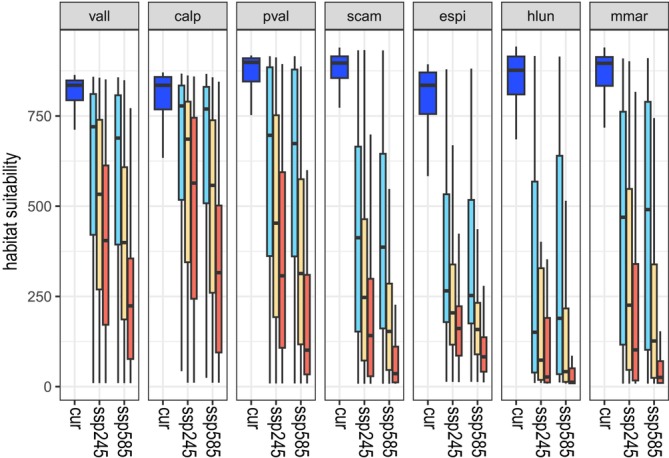
Boxplots of the predicted habitat suitability values for occurrences of the seven species at different time periods (blue = current, cyano = 2021–2040, orange = 2041–2060 and red = 2061–2080) under different climate change scenarios (ssp245 and ssp585). Species abbreviations are: Vall = *Veronica allionii*, calp = *Campanula alpestris*, pval = *Potentilla valderia*, scam = *Silene campanula*, espi = *Eryngium spinalba*, hlun = *Helianthemum lunulatum* and mmar = *Micromeria marginata*. Species are sorted according to elevation centre.

**FIGURE 3:**
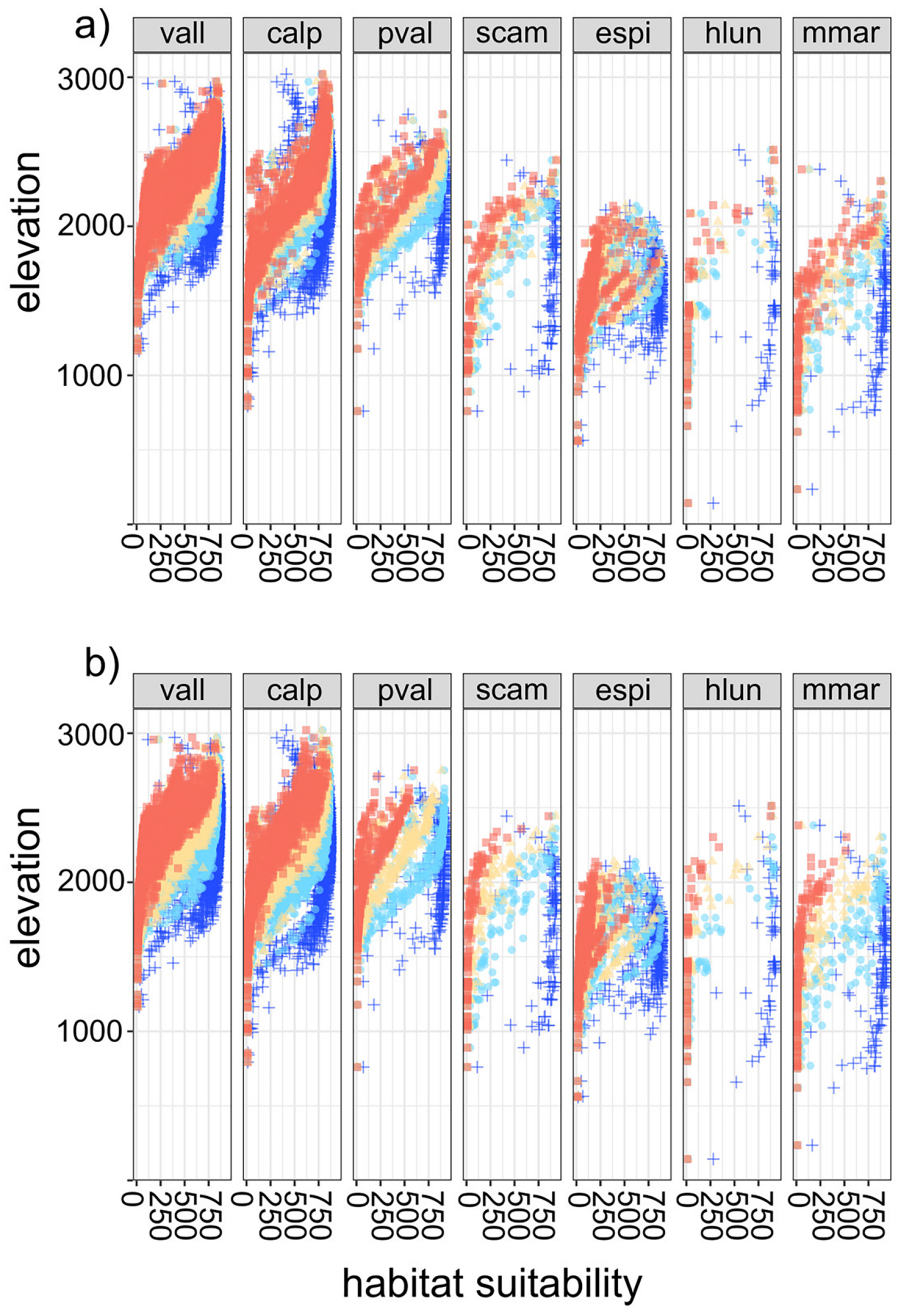
Boxplots of the predicted habitat suitability values according to elevation of known localities of the seven species at different time periods (violet = current, orange = 2021–2040, green = 2041–2060 and cyano = 2061–2080) under (a) ssp245 and (b) ssp585 climate change scenario. Species abbreviations are: All = Veronica allionii, calp = Campanula alpestris, pval = Potentilla valderia, scam = Silene campanula, espi = Eryngium spinalba, hlun = Helianthemum lunulatum and mmar = Micromeria marginata. Species are sorted according to elevation centre.

Under current climate, model evaluation metrics (i.e., AUC and TSS) indicated good model performance for all modelling techniques and species (Table 1). Considering all species together, we found differences between the two future scenarios in the trend of habitat suitability (HS) reduction in current occurrences mainly between far time slices (Figure 2). Habitat suitability decreases very suddenly and dramatically in the three species growing on cliffs (i.e., *H. lunulatum, M. marginata* and *S. campanula* ) and in one of the species growing on grassland (i.e., *E. spinalba*), while in the other species the reduction is observed starting from 2041 to 2060 (Figure 2). The decrease in habitat suitability is weak in *C. alpestris* (in the period 2061 to 2080 the mean HS is 485.6 and 313.3 in SSP245 and SSP585, respectively) and *P. valderia* (in the period 2061 to 2080 the mean HS is 406.4 and 249.7 in SSP245 and SSP585, respectively). Differently, a drastic reduction in habitat suitability values occurs in *E. spinalba* and *H. lunulatum*, for which in the period 2061 to 2080 mean HS values decrease below 200 in the SSP245 and below 100 in SSP585 (Figure 2). Some low‐elevation species, such as *H. lunulatum*, *S. campanula* and *M. marginata* , will gain HS at high elevations and lose it elsewhere in their range (Figure 3). Differently, the other low‐elevation species *E. spinalba* is projected to lose HS mainly at low elevation. Among high‐elevation species, *C. alpestris* and *V. allionii* are predicted to increase their HS at high altitude and lose little at low altitude, while *P. valderia* will increase little HS at the highest elevation but its HS is forecasted to decrease dramatically at lower elevation (Figure 3).

**TABLE 1:** Model performance measured using the area under the curve (AUC) of the relative operating characteristic curve and the true skill statistic (TSS).

	GBM	GLM	MARS	SRE	MaxNet
*C. alpestris*					
AUC	0.937 (0.005)	0.931 (0.005)	0.933 (0.005)	0.871 (0.007)	0.933 (0.005)
TSS	0.772 (0.013)	0.762 (0.013)	0.764 (0.015)	0.743 (0.015)	0.764 (0.014)
*E. spinalba*					
AUC	0.948 (0.007)	0.896 (0.008)	0.938 (0.008)	0.849 (0.013)	0.942 (0.008)
TSS	0.791 (0.020)	0.679 (0.019)	0.768 (0.024)	0.699 (0.026)	0.771 (0.024)
*H. lunulatum*					
AUC	0.944 (0.043)	0.979 (0.008)	0.972 (0.022)	0.909 (0.050)	0.982 (0.009)
TSS	0.682 (0.124)	0.899 (0.049)	0.843 (0.088)	0.816 (0.100)	0.908 (0.052)
*M. marginata*					
AUC	0.973 (0.017)	0.985 (0.003)	0.980 (0.043)	0.928 (0.029)	0.986 (0.003)
TSS	0.858 (0.053)	0.917 (0.033)	0.904 (0.086)	0.856 (0.057)	0.919 (0.031)
*P. valderia*					
AUC	0.979 (0.010)	0.986 (0.003)	0.986 (0.005)	0.928 (0.018)	0.988 (0.002)
TSS	0.911 (0.023)	0.923 (0.017)	0.924 (0.019)	0.857 (0.036)	0.920 (0.019)
*S. campanula*					
AUC	0.978 (0.017)	0.989 (0.005)	0.976 (0.065)	0.927 (0.031)	0.991 (0.003)
TSS	0.850 (0.061)	0.919 (0.029)	0.916 (0.080)	0.855 (0.062)	0.922 (0.034)
*V. allionii*					
AUC	0.944 (0.003)	0.926 (0.004)	0.930 (0.004)	0.874 (0.007)	0.945 (0.003)
TSS	0.806 (0.011)	0.788 (0.009)	0.792 (0.010)	0.749 (0.014)	0.807 (0.010)

*Note:* The values represent the mean values of evaluation runs for each algorithm; values in brackets indicate standard deviation.

**FIGURE 4:**
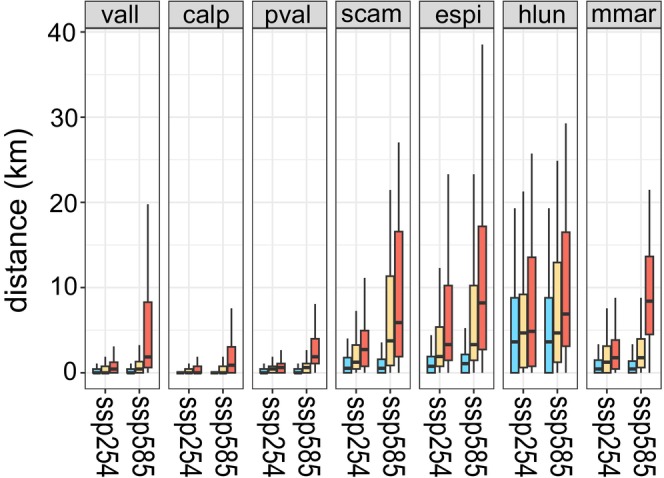
Boxplots showing the distance in Km between current occurrences and closest suitable areas for the seven studied species at different time periods (cyano = 2021–2040, orange = 2041–2060 and red = 2061–2080) under different climate change scenario (ssp245 and ssp585). Species abbreviations are: Vall = *Veronica allionii*, calp = *Campanula alpestris*, pval = *Potentilla valderia*, scam = *Silene campanula*, espi = *Eryngium spinalba*, hlun = *Helianthemum lunulatum* and mmar = *Micromeria marginata*. Species are sorted according to elevation centre.

In general, the distance from current occurrences to closest suitable areas in the future increases with time and is higher in SSP585 than in SSP245 (Figure 4). In *H. lunulatum* the distance increases sharply since the first time slice for both scenarios and this species has to move on average more than 4–5 km in furthest times. In SSP245, populations of *P. valderia*, *V. allionii* and *C. alpestris* have to move on average from a few hundred meters in the first time period to a few thousand meters in the last time periods to meet the closest suitable areas. Differently, in SSP585, for populations of *P. valderia* and *V. allionii*, two species growing on grasslands, a distance from a few hundred to a few thousand meters is enough to meet the closest suitable area according to the time slice. On the contrary, in the case of *C. alpestris* , the distance remains always within the range of a few hundred meters, even in the worst scenario (Figure 4). In *S. campanula* and *E. spinalba* the distance increases gradually over time and in *M. marginata* the distance increases sharply only between the two most distant time periods in SSP585.

**FIGURE 5:**
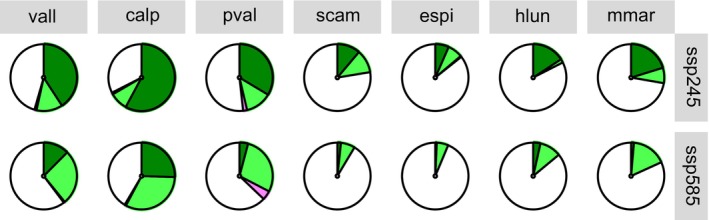
Pie chart showing the percentage of long‐term refugia (green), holdouts (light green) and stepping stones (pink) with respect to the occurrences of the seven studied species under different climate change scenarios (ssp245 and ssp585). Blank represents populations outside any refugia type. Species abbreviations are: Vall = *Veronica allionii*, calp = *Campanula alpestris*, pval = *Potentilla valderia*, scam = *Silene campanula*, espi = *Eryngium spinalba*, hlun = *Helianthemum lunulatum* and mmar = *Micromeria marginata*. Species are sorted according to elevation centre.

The percentage of populations growing in refugia is lower in the three species growing on screes (*H. lunulatum*, *S. campanula* and *M. marginata* ) and in one of the species from grasslands (*E. spinalba*; Figure 5). Differently, in *C. alpestris* and *V. allionii* under the SSP245 scenario, the percentage of refugial areas exceeds the percentage of populations outside any refugia type. In particular, the populations falling outside any type of refugia range from 33% to 86% in SSP254 and from 42% to 94% in SSP585 (in *C. alpestris* and *E. spinalba*, respectively). The percentage of refugia decreases as the climatic conditions worsen (Figure 5). In fact, the percentage of populations falling in refugia ranges from 7% to 58% in SSP254 and from 0.7% to 25% in SSP585 (in *E. spinalba* and *C. alpestris* , respectively). Holdouts are generally less frequent under the SSP245 scenario, ranging from 1.75% to 13% (in *H. lunulatum* and *P. valderia*, respectively). Differently, under the SSP585 scenario, the percentage of holdouts is higher than the percentage of other refugia types in all species and ranges from 6% to 32% (in *E. spinalba* and *C. alpestris* , respectively). Stepping stones are the rarest persistence areas under both scenarios, ranging from nearly 0% in both scenarios for the three species of the cliffs and for *E. spinalba* to 2% and 4.5% for *P. valderia* in SSP245 and SSP585, respectively (Figure 5). Moreover, only high elevation species (*V. allionii*, *C. alpestris* and *P. valderia*) have stepping stones under both climate scenarios.

**FIGURE 6:**
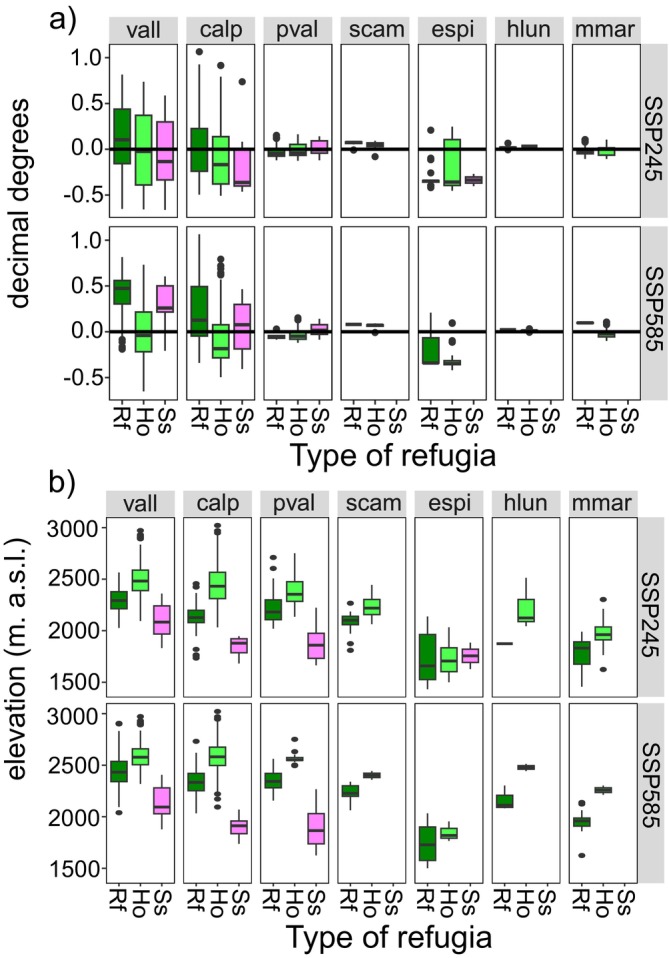
Boxplots showing the location of long‐term refugia (green), holdouts (light green) and stepping stones (pink) with respect to (a) mean latitude and (b) elevation within the range of distribution of the seven studied species under different climate change scenario (ssp245 and ssp585). Latitude values are given positive or negative in relation to zero which represent the median latitude of the populations in the current time. Species abbreviations are: Vall = *Veronica allionii*, calp = *Campanula alpestris*, pval = *Potentilla valderia*, scam = *Silene campanula*, espi = *Eryngium spinalba*, hlun = *Helianthemum lunulatum* and mmar = *Micromeria marginata*. Species are sorted according to elevation centre.

The different types of refugia are generally a little further south than the barycentre of species ranges (Figure 6a). Stepping stones and holdouts are southernmost in *C. alpestris* and stepping stones in *V. allionii* in SSP245. A northward shift is detected only for refugia and stepping stones of *C. alpestris* and *V. allionii* in SSP585. Moreover, holdouts are located in the upper part of species' elevation range, refugia in the middle part and stepping stones, when present, in the lower part (Figure 6b). The only exception is *E. spinalba*, for which all refugia types are at the same elevation.

## 4 Discussion

The effect of climate change on currently known populations of the studied species seems to be highly idiosyncratic. Four out of seven species are forecasted to experience a rapid and severe decline in the habitat conditions (Figure 2), whereas the remaining species are expected to experience a severe, but more gradual impact. Furthermore, even if the effect is generally more dramatic under harsher climatic conditions (i.e., SSP585), in *H. lunulatum* the difference between the two scenarios is very weak. Despite differences among species, the impact of climate change is lower on high‐elevation species (i.e., *C. alpestris, P. valderia* and *V. allionii*) than in species belonging to the montane belt (i.e., *E. spinalba*, *H. lunulatum*, *S. campanula* and *M. marginata* ), as previously detected in other studies (Engler et al. 2011; Twomey et al. 2012; Gibson‐Reinemer and Rahel 2015; Dagnino et al. 2020). Furthermore, the change in habitat suitability across the elevational range is species‐specific, with some species increasing habitat suitability values at high elevations and losing them at low elevations, others losing habitat suitability across the entire elevation range, and others having a weak loss at low elevations. In general, habitat suitability is expected to increase at a higher elevation and to decline at low elevation because of the increase in temperature (Vitasse et al. 2021). Along the altitudinal gradient, however, the pattern of temperature change can be or not concordant with that of precipitation (Beniston 2006; Lionello and Scarascia 2018). For example, in Mediterranean mountains although temperature is increasing wherever, it was observed a positive precipitation trend only in the mountain belt (< 1500 m a.s.l.), while a negative or no trend was observed in the alpine belt (Sigro et al. 2024). As water availability is likely to be the main determinant of range dynamics mainly at low elevation (Martínez‐Vilalta et al. 2023), this precipitation pattern may mitigate habitat suitability loss at low elevation. Therefore, species may respond consistently to their optimum climatic conditions persisting in wet areas located at low elevation. In fact, species such as *C. alpestris* and *V. allionii* maintain similar levels of habitat suitability to the current in the lower part of their elevation range (below 1500 m a.s.l.). The inconsistency of population responses to climate change across species highlights how specific environmental requirements can often affect the timing and magnitude of the effects of changing climate.

Our results suggest that in situ refugia are the most frequent refugia type in the most optimistic climatic scenario, while holdouts are the most frequent in the pessimistic one (Figure 5). This result supports the idea that if climate change falls within the limits already experienced by species during the Holocene (as projected under the SSP245 scenario; Guiot and Cramer 2016; Fauquette et al. 2018), several populations of the species will be able to survive in situ. Stepping stones are very rare under both climates suggesting that endemics will not be likely able to track change in future climate regardless of the magnitude of the change. However, unlike the other species analysed which are poor dispersers, *E. spinalba* is endozoochorous and can disperse over long distances (from hundreds to thousands of metres; Lososová et al. 2023). Then, for this species the percentage of stepping stones may be underestimated, even if its rarity and the low population size make it more unlikely for long‐distance dispersal events. Despite their rarity, a previous study suggests that stepping stones, together with climatically suitable area, have played an important role in ensuring the survival of SW Alps endemics over time (Patsiou et al. 2014; Casazza, Barberis, et al. 2016). Moreover, in our study, stepping stones totally lacked in low elevation species (i.e., *H. lunulatum, M. marginata* and *S. campanula* ). This result, together with the greater distance of the nearest suitable area in mountain species (Figure 4), supports the idea that the lower the range limits and climatic optima were situated historically, the faster they are expected to shift upwards (Rumpf et al. 2018).

In general, centres of endemism occur where past climatic fluctuations have been weakened by rough topography and microclimatic diversity, and likely these areas will remain relatively climatically buffered also in the future (Harrison and Noss 2017). However, the reduction of the persistence area quality (i.e., from refugia to holdouts) under the studied scenarios suggests that the strength of the buffering capability is highly dependent on the magnitude of climate change.

All refugia types were generally located in the South‐central part of the species distribution range (Figure 6) corresponding to the highest peaks of the Maritime and Ligurian Alps—an area that in the past acted as refugia for many endemics (Casazza, Grassi, et al. 2016). This result is in line with the previous finding that hotspots of climate change are widespread in the Alps and that coldspots occur mainly in peripheral areas, such as the Maritime and Ligurian Alps (Lai et al. 2022). Moreover, we detected an altitudinal displacement of refugia types with low capacity refugia at low elevation and high capacity refugia at high elevation (Figure 6). Taken together these results suggest that cold‐tolerant taxa will survive at high altitudes rather than at high latitudes. This depends on the amplitude of impact of a warming climate on these species which is mainly determined by mountain average elevations. The southern region of the Alps is experiencing moderate warming in winter compared with the other parts and an increase in wet days because of changes in atmospheric circulation (Gobiet et al. 2014). Similarly, the maximum warming in winter and spring will occur between 1000 and 2000 m a.s.l., while in summer and autumn the maximum warming will shift upwards, slightly above 2000 m a.s.l. (Kotlarski et al. 2023). In line with climatic patterns, we detected stepping stones mainly between 1500 and 2000 m a.s.l., holdouts above 2000 m and refugia at around 2500 m a.s.l. (Figure 6).

According to our results, refugia showed different capacity for facilitating the persistence of the studied populations. Notably, in all species included in this study, the capacity of refugia changes over the species distributional range, supporting the idea that conservation plans must account for these changes occurring in the different parts of the species range (Hannah et al. 2014; Keppel and Wardell‐Johnson 2015). In particular, at low elevations, where most of the stepping stones are located, the degree of anthropisation and resulting habitat fragmentation are likely to be higher. For these populations, it will be important to assure the connectivity of patches so that individuals may move on tracking newly suitable areas. Refugia that are located at intermediate altitudes might assure long‐term persistence of populations. Unfortunately, in the Western Alps, these areas are also prone to habitat change for the expansion of the forests causing the reduction of grasslands where several endemics occur. In these cases, the management of the habitat (subalpine seminatural grasslands) will play a pivotal role in ensuring the effectiveness of climatic refugia. Populations growing in holdouts, that assure survival for only short time periods, should be considered for assisted colonisation, possibly in suitable areas within protected areas (Casazza et al. 2021). Overall, our results support the idea that the assessment of refugial quality allows a shift from place‐based to process‐based conservation strategies (Hannah et al. 2014).

## Author Contributions

**Gabriele Casazza:** conceptualization (lead), data curation (supporting), formal analysis (equal), methodology (lead), supervision (equal), validation (equal), visualization (lead), writing – original draft (lead). **Martino Adamo:** data curation (equal), formal analysis (equal), methodology (equal), writing – review and editing (equal). **Maria Guerrina:** conceptualization (lead), data curation (equal), methodology (equal), visualization (equal). **Luigi Minuto:** conceptualization (equal), supervision (equal). **Marco Mucciarelli:** funding acquisition (lead), methodology (equal), project administration (lead), supervision (equal), visualization (equal), writing – review and editing (lead).

## Funding

This work was supported by the NextGenerationEU, CN_00000033.

## Conflicts of Interest

The authors declare no conflicts of interest.

## Data Availability

All species included in the study are protected by European National or Regional laws (see Material and Methods section). All data are shared at a 5 × 5 km resolution. Species occurrences are available at the doi link https://doi.org/10.6084/m9.figshare.29224586. At the same link, we made publicly available the geotiff raster files of the predictions of each species.

## References

[ece372555-bib-0001] Allouche, O. , A. Tsoar , and R. Kadmon . 2006. “Assessing the Accuracy of Species Distribution Models: Prevalence, Kappa and the True Skill Statistic (TSS).” Journal of Applied Ecology 43: 1223–1232. 10.1111/j.13652664.2006.01214.x.

[ece372555-bib-0002] Araújo, M. B. , R. P. Anderson , A. Márcia Barbosa , et al. 2019. “Standards for Distribution Models in Biodiversity Assessments.” Science Advances 5: eaat4858. 10.1126/sciadv.aat4858. 30746437 PMC6357756

[ece372555-bib-0003] Araújo, M. B. , and M. New . 2007. “Ensemble Forecasting of Species Distributions.” Trends in Ecology & Evolution 22: 42–47. 10.1016/j.tree.2006.09.010. 17011070

[ece372555-bib-0004] Baselga, A. , J. M. Lobo , J. C. Svenning , and M. B. Araújo . 2012. “Global Patterns in the Shape of Species Geographical Ranges Reveal Range Determinants.” Journal of Biogeography 39: 760–771. 10.1111/j.1365-2699.2011.02612.x.

[ece372555-bib-0005] Baumgartner, J. B. , M. Esperón‐Rodríguez , and L. J. Beaumont . 2018. “Identifying In Situ Climate Refugia for Plant Species.” Ecography 41: 1850–1863. 10.1111/ecog.03431.

[ece372555-bib-0006] Beniston, M. 2006. “Mountain Weather and Climate: A General Overview and a Focus on Climatic Change in the Alps.” Hydrobiologia 562: 3–16. 10.1007/s10750-005-1802-0.

[ece372555-bib-0146] Casazza, G. , T. Abeli , G. Bacchetta , et al. 2021. “Combining Conservation Status and Species Distribution Models for Planning Assisted Colonisation Under Climate Change.” Journal of Ecology 109, no. 6: 2284–2295. 10.1111/1365-2745.13606.

[ece372555-bib-0007] Casazza, G. , G. Barberis , M. Guerrina , E. Zappa , M. Mariotti , and L. Minuto . 2016. “The Plant Endemism in the Maritime and Ligurian Alps.” Biogeographia 31: 73–88. 10.21426/B631132738.

[ece372555-bib-0008] Casazza, G. , F. Grassi , G. Zecca , and L. Minuto . 2016. “Phylogeographic Insights Into a Peripheral Refugium: The Importance of Cumulative Effect of Glaciation on the Genetic Structure of Two Endemic Plants.” PLoS One 11: e0166983. 10.1371/journal.pone.0166983. 27870888 PMC5117763

[ece372555-bib-0009] Chytrý, K. , N. Helm , K. Hülber , et al. 2024. “Limited Impact of Microtopography on Alpine Plant Distribution.” Ecography 2024: e06744. 10.1111/ecog.06744.

[ece372555-bib-0010] Dagnino, D. , M. Guerrina , L. Minuto , M. G. Mariotti , F. Médail , and G. Casazza . 2020. “Climate Change and the Future of Endemic Flora in the South Western Alps: Relationships Between Niche Properties and Extinction Risk.” Regional Environmental Change 20: 121. 10.1007/s10113-020-01708-4.

[ece372555-bib-0011] Engler, R. , C. F. Randin , W. Thuiller , et al. 2011. “21st Century Climate Change Threatens Mountain Flora Unequally Across Europe.” Global Change Biology 17: 2330–2341. 10.1111/j.1365-2486.2010.02393.x.

[ece372555-bib-0012] Essl, F. , M. Staudinger , O. Stöhr , L. Schratt‐Ehrendorfer , W. Rabitsch , and H. Niklfeld . 2009. “Distribution Patterns, Range Size and Niche Breadth of Austrian Endemic Plants.” Biological Conservation 142: 2547–2558. 10.1016/j.biocon.2009.05.027.

[ece372555-bib-0013] Fauquette, S. , J.‐P. Suc , F. Médail , et al. 2018. “The Alps: A Geological, Climatic, and Human Perspective on Vegetation History and Modern Plant Diversity.” In Mountains, Climate, and Biodiversity, edited by C. Hoorn , A. Perrigo , and A. Antonelli , 413–428. Wiley.

[ece372555-bib-0014] Gibson‐Reinemer, D. K. , and F. J. Rahel . 2015. “Inconsistent Range Shifts Within Species Highlight Idiosyncratic Responses to Climate Warming.” PLoS One 10: e0132103. 10.1371/journal.pone.0132103. 26162013 PMC4498742

[ece372555-bib-0015] Gillett, N. P. 2024. “Halving of the Uncertainty in Projected Warming Over the Past Decade.” NPJ Climate and Atmospheric Science 7: 146. 10.1038/s41612-024-00693-3.

[ece372555-bib-0016] Gobiet, A. , S. Kotlarski , M. Beniston , G. Heinrich , J. Rajczak , and M. Stoffel . 2014. “21st Century Climate Change in the European Alps: A Review.” Science of the Total Environment 493: 1138–1151. 10.1016/j.scitotenv.2013.07.050. 23953405

[ece372555-bib-0017] Guiot, J. , and W. Cramer . 2016. “Climate Change: The 2015 Paris Agreement Thresholds and Mediterranean Basin Ecosystems.” Science 354: 465–468. 10.1126/science.aah5015. 27789841

[ece372555-bib-0018] Guisan, A. , and N. E. Zimmermann . 2000. “Predictive Habitat Distribution Models in Ecology.” Ecological Modelling 135: 147–186. 10.1016/S0304-3800(00)00354-9.

[ece372555-bib-0019] Hanley, J. A. , and B. J. McNeil . 1982. “The Meaning and Use of the Area Under a Receiver Operating Characteristic (ROC) Curve.” Radiology 143: 29–36. 10.1148/radiology.143.1.7063747. 7063747

[ece372555-bib-0020] Hannah, L. , L. Flint , A. Syphard , et al. 2014. “Fine‐Grain Modeling of Species' Response to Climate Change: Holdouts, Stepping‐Stones, and Microrefugia.” Trends in Ecology & Evolution 29: 390–397. 10.1016/j.tree.2014.04.006. 24875589

[ece372555-bib-0021] Harrison, S. , and R. Noss . 2017. “Endemism Hotspots Are Linked to Stable Climatic Refugia.” Annals of Botany 119, no. 2: 207–214. 10.1093/aob/mcw248. 28064195 PMC5321063

[ece372555-bib-0022] Karger, D. N. , O. Conrad , J. Böhner , et al. 2017. “Climatologies at High Resolution for the Earth Land Surface Areas.” Scientific Data 4: 170122. 10.1038/sdata.2017.122. 28872642 PMC5584396

[ece372555-bib-0023] Keppel, G. , and G. W. Wardell‐Johnson . 2015. “Refugial Capacity Defines Holdouts, Microrefugia and Stepping‐Stones: A Response to Hannah Et al.” Trends in Ecology & Evolution 30: 233–234. 10.1016/j.tree.2015.01.008. 25683027

[ece372555-bib-0024] Kotlarski, S. , A. Gobiet , S. Morin , M. Olefs , J. Rajczak , and R. Samacoïts . 2023. “21st Century Alpine Climate Change.” Climate Dynamics 60: 65–86. 10.1007/s00382-022-06303-3.

[ece372555-bib-0025] Lai, Q. , S. Hoffmann , A. Jaeschke , and C. Beierkuhnlein . 2022. “Emerging Spatial Prioritization for Biodiversity Conservation Indicated by Climate Change Velocity.” Ecological Indicators 138: 108829. 10.1016/j.ecolind.2022.108829.

[ece372555-bib-0026] Lenoir, J. , J. C. Gégout , P. A. Marquet , et al. 2008. “A Significant Upward Shift in Plant Species Optimum Elevation During the 20th Century.” Science 320: 1768–1771. 10.1126/science.1156831. 18583610

[ece372555-bib-0027] Lenoir, J. , T. Hattab , and G. Pierre . 2017. “Climatic Microrefugia Under Anthropogenic Climate Change: Implications for Species Redistribution.” Ecography 40: 253–266. 10.1111/ecog.02788.

[ece372555-bib-0028] Lionello, P. , and L. Scarascia . 2018. “The Relation Between Climate Change in the Mediterranean Region and Global Warming.” Regional Environmental Change 18: 1481–1493. 10.1007/s10113-018-1290-1.

[ece372555-bib-0029] Lososová, Z. , I. Axmanová , M. Chytrý , et al. 2023. “Seed Dispersal Distance Classes and Dispersal Modes for the European Flora.” Global Ecology and Biogeography 32: 1485–1494. 10.1111/geb.13712.

[ece372555-bib-0030] Manes, S. , M. J. Costello , H. Beckett , et al. 2021. “Endemism Increases Species' Climate Change Risk in Areas of Global Biodiversity Importance.” Biological Conservation 257: 109070. 10.1016/j.biocon.2021.109070.

[ece372555-bib-0032] Martínez‐Vilalta, J. , R. García‐Valdés , A. Jump , A. Vilà‐Cabrera , and M. Mencuccini . 2023. “Accounting for Trait Variability and Coordination in Predictions of Drought‐Induced Range Shifts in Woody Plants.” New Phytologist 240: 23–40. 10.1111/nph.19138. 37501525

[ece372555-bib-0033] Michalak, J. L. , J. J. Lawler , D. R. Roberts , and C. Carroll . 2018. “Distribution and Protection of Climatic Refugia in North America.” Conservation Biology 32: 1414–1425. 10.1111/cobi.13130. 29744936

[ece372555-bib-0034] Montràs‐Janer, T. , A. J. Suggitt , R. Fox , et al. 2024. “Anthropogenic Climate and Land‐Use Change Drive Short‐ and Long‐Term Biodiversity Shifts Across Taxa.” Nature Ecology & Evolution 8: 739–751. 10.1038/s41559-024-02326-7. 38347088 PMC11009105

[ece372555-bib-0035] Morgan, J. W. , and S. E. Venn . 2017. “Alpine Plant Species Have Limited Capacity for Long‐Distance Seed Dispersal.” Plant Ecology 218: 813–819. http://www.jstor.org/stable/26165318.

[ece372555-bib-0036] Mouillot, D. , D. R. Bellwood , B. Christopher , et al. 2013. “Rare Species Support Vulnerable Functions in High‐Diversity Ecosystems.” PLoS Biology 11: 1–11. 10.1371/journal.pbio.1001569 PMC3665844 23723735

[ece372555-bib-0037] Patsiou, T. S. , E. Conti , N. E. Zimmermann , S. Theodoridis , and C. F. Randin . 2014. “Topo‐Climatic Microrefugia Explain the Persistence of a Rare Endemic Plant in the Alps During the Last 21 Millennia.” Global Change Biology 20: 2286–2300. 10.1111/gcb.12515. 24375923

[ece372555-bib-0038] Petitpierre, B. , O. Broennimann , C. Kueffer , C. Daehler , and A. Guisan . 2017. “Selecting Predictors to Maximize the Transferability of Species Distribution Models: Lessons From Cross‐Continental Plant Invasions.” Global Ecology and Biogeography 26: 275–287. 10.1111/geb.12530.

[ece372555-bib-0039] Rumpf, S. B. , K. Hülber , G. Klonner , et al. 2018. “Range Dynamics of Mountain Plants Decrease With Elevation.” Proceedings of the National Academy of Sciences of the United States of America 115: 1848–1853. 10.1073/pnas.1713936115. 29378939 PMC5828587

[ece372555-bib-0040] Sigro, J. , M. Cisneros , A. J. Perez‐Luque , C. Perez‐Martinez , and T. Vegas‐Vilarrubia . 2024. “Trends in Temperature and Precipitation at High and Low Elevations in the Main Mountain Ranges of the Iberian Peninsula (1894–2020): The Sierra Nevada and the Pyrenees.” International Journal of Climatology 44: 2897–2920. 10.1002/joc.8487.

[ece372555-bib-0041] Thuiller, W. , B. Lafourcade , R. Engler , and M. B. Araújo . 2009. “BIOMOD—A Platform for Ensemble Forecasting of Species Distributions.” Ecography 32: 369–373.

[ece372555-bib-0042] Thuiller, W. , S. Lavergne , C. Roquet , I. Boulangeat , B. Lafourcade , and M. B. Araujo . 2011. “Consequences of Climate Change on the Tree of Life in Europe.” Nature 470: 531–534. 10.1038/nature09705. 21326204

[ece372555-bib-0043] Thuiller, W. , G. F. Midgley , G. O. Hughes , et al. 2006. “Endemic Species and Ecosystem Sensitivity to Climate Change in Namibia.” Global Change Biology 12: 759–776. 10.1111/j.1365-2486.2006.01140.x.

[ece372555-bib-0044] Twomey, M. , E. Brodte , U. Jacob , U. Brose , T. P. Crowe , and M. C. Emmerson . 2012. “Idiosyncratic Species Effects Confound Size‐Based Predictions of Responses to Climate Change.” Philosophical Transactions of the Royal Society, B: Biological Sciences 367: 2971–2978. 10.1098/rstb.2012.0244. PMC3479753 23007085

[ece372555-bib-0045] Vitasse, Y. , S. Ursenbacher , G. Klein , et al. 2021. “Phenological and Elevational Shifts of Plants, Animals and Fungi Under Climate Change in the European Alps.” Biological Reviews 96: 1816–1835. 10.1111/brv.12727. 33908168

[ece372555-bib-0046] Zurell, D. , J. Franklin , C. König , et al. 2020. “A Standard Protocol for Reporting Species Distribution Models.” Ecography 43: 1261–1277. 10.1111/ecog.04960.

